# Supplementary material for: Production and Characterisation of Stabilised PV-3 Virus-like Particles Using Pichia pastoris
Source: Viruses. 2022 Sep 30;14(10):2159. doi: 10.3390/v14102159 (PMC9611624; doi:10.3390/v14102159)

Supplementary Information

Supplementary Table S1. Cryo-EM data collection and image processing for PV-3 SC8

**Parameter**	**Value**
**Data Collection**	
Voltage (kV)	300
Magnification (×)	60975
Defocus range (μm)	-2.4 to -0.9
Dose rate (e^-/pixel/s)	9.20
Frames	20
Frame length (s)	0.150
Total electron dose (e^-/Å^2)	35.06
Micrographs	3717
**Data processing**	
Pixel size (Å)	0.82
Initial particles (no.)	10744
Final particles (no.)	2712
Box size (pixels)	450
Symmetry	I1
Accuracy of rotations (°)	0.1320
Accuracy of translations (Å)	0.2662
Resolution (Å)	2.7
Map sharpening *B*-factor (Å^2)	-59.1

Supplementary Table S2. Structure refinement and validation for the PV-3 SC8 capsid protein

**Parameter**	**Value**
**Model composition**	
Non-hydrogen atoms	5652
Protein residues	712
Ligands	Sphingosine:1
**Refinement**	
Resolution (Å)	2.7
Map CC[a] (Mask)	0.85
Map CC[a] (Volume)	0.81
**RMS deviations**	
Bond lengths (Å)	0.002
Bond angles (°)	0.502
**Mean B-factor ($Å^2$)**	
Protein	13.05
Ligand	7.92
**Validation**	
Molprobity[b] score (percentile)	1.22 (99th)
Clashscore[b], all atoms (percentile)	3.67 (97th)
Ramachandran favoured (%)	97.71
Ramachandran allowed (%)	2.29
Ramachandran outliers (%)	0.00
Rotamer favoured (outliers) (%)	95.03 (0.32)
Cβ deviations >0.25 Å (%)	0.00
CaBLAM outliers (%)	1.20
CA Geometry outliers (%)	0.44
**EMRinger[c] score**	4.63

[a] Map CC is given for the full particle reconstruction.

[b] Chen *et al.* (2010) Acta Crystallographica D66:12-21.

[c] Barad *et al.* (2015) Nature Methods 12:943–946.

Supplementary Figure S1. Resolution of the PV-3 SC8 cryo-EM reconstruction. A Fourier shell correlation (FSC) calculated between two independent half sets of data as a function of spatial frequency is plotted for the PV-3 SC8 cryo-EM reconstruction. FSC is plotted for the original unmasked half-maps (grey) and masked half-maps that had density corresponding to solvent removed (blue). FSC is also shown for phase-randomized half-maps (red) used to compensate for possible effects of the masking procedure before calculating the final corrected FSC (black). Good agreement between the masked and corrected curves indicated no adverse effects from the masking. The resolution at which the corrected curve drops below the FSC=0.143 threshold (grey dashed line) is indicated with an arrow. **B** Local resolution analysis of the final cryo-EM electron potential map for PV-3 SC8 as assessed by RELION local resolution estimation. A central slice through the VLP is viewed along the 2-fold axis and the distribution of local resolution is shown coloured from blue to red according to the colour key shown.

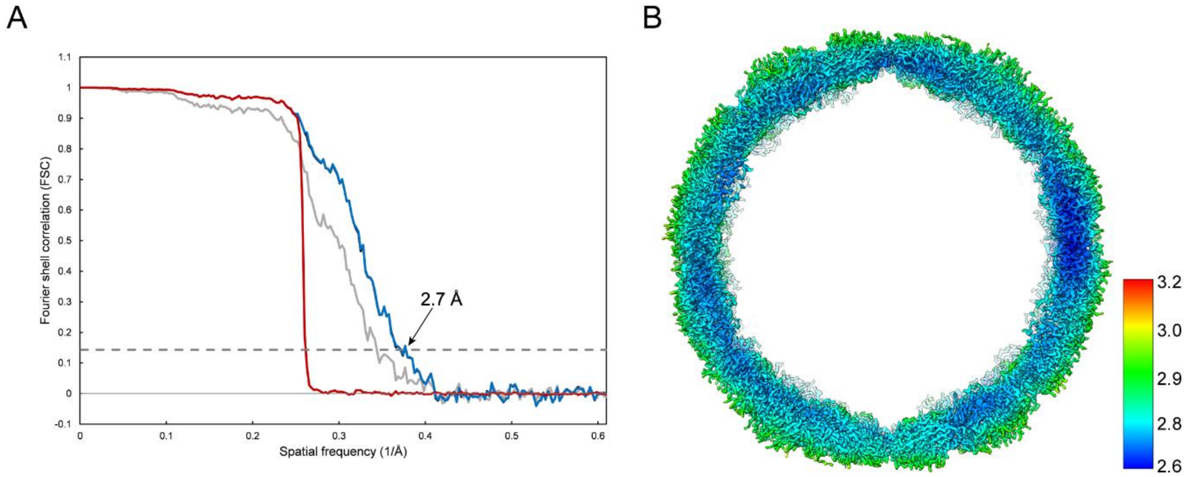

Supplement: Supplementary file 1 [file viruses-14-02159-s001.zip › viruses-1894136-supplementary.pdf]
